# Structural vulnerabilities and HIV risk among sexual minority female sex workers (SM-FSW) by identity and behavior in Baltimore, MD

**DOI:** 10.1186/s12954-020-00383-2

**Published:** 2020-06-15

**Authors:** Jennifer L. Glick, Sahnah Lim, S. Wilson Beckham, Catherine Tomko, Ju Nyeong Park, Susan G. Sherman

**Affiliations:** 1 Department of Health Behavior and Society, Johns Hopkins Bloomberg School of Public Health, Baltimore, MD 21205 USA; 2 Department of Population Health, NYU School of Medicine, New York, NY USA

**Keywords:** Vulnerability, Sexual minority women (SMW), Female sex workers (FSW), HIV risk

## Abstract

**Background:**

Research suggests sexual minority female sex workers (SM-FSW) face elevated structural vulnerability and HIV risk compared to their heterosexual counterparts. Structural vulnerabilities reflect societal level factors (e.g., sexism, homophobia, racism) that constrain an individual’s agency, particularly related to health outcomes. This study examines the association between SM status by identity and behavior, structural vulnerability, and HIV risk among a sample of street-based FSW.

**Methods:**

The current study utilizes baseline data from the SAPPHIRE study, a prospective cohort of cis gender and transgender FSW in Baltimore, MD, recruited through targeted time-location sampling from April 2016 to January 2017. The current analysis focuses on cisgender women. The baseline survey ascertained demographics, substance use, intimate partner violence (IPV), and sex work characteristics. Multivariable models were constructed using self-identity and behaviorally defined SM status as independent variables with vulnerability outcomes (e.g., injection drug use, injection speedball, binge drinking, homelessness, physical IPV, ever had a pimp, and being a minor at sex work entry (age < 18)) as dependent variables.

**Results:**

Of the participants (*n* = 247), 25.5% (*n* = 63) self-identified as a SM by identity (e.g., gay or bisexual), and 8.5% (*n* = 21) reported SM behavior (e.g., same-gender sexual behavior) in the past 3 months. In multivariable logistic regression models, SM status by identity was associated with increased odds of injection drug use, binge drinking, homelessness, physical IPV, and being a minor at sex work entry. SM status by behavior was associated with increased odds of binge drinking, homelessness, ever having a pimp, and being a minor at sex work entry.

**Conclusion:**

The study indicates disproportionate structural vulnerability and heightened HIV risk among SM-FSW, as compared to their heterosexual counterparts, with differences in their profile by sexual identity and behavior. Findings suggest a need for nuanced interventions tailored to these populations.

## Background

Female sex workers (FSW) who are also sexual minorities (SM-FSW) face greater structural vulnerabilities, which exacerbate their HIV risk, compared to their heterosexual counterparts [1–4]. Structural vulnerabilities reflect societal-level factors (e.g., sexism, homophobia, racism) that constrain an individual’s agency, particularly related to health outcomes [5]. While studies about HIV risk and SM-FSW are limited, data indicate that sexual minority women (SMW) are more likely to engage in sex work and experience disparate amounts of structural vulnerability (e.g., housing insecurity, violence) and therefore have worse HIV risk outcomes than their heterosexual counterparts [2–4].

Current best practices on conceptualizing and measuring sexual orientation incorporate a three dimensional model including identity, behavior, and attraction [6, 7]. Identity is self-defined based on how one identifies their sexual orientation. Behavior concerns the gender of sexual partners in relation to one’s own gender. Attraction concerns the gender of those whom one is sexually attracted to, in relation to one’s own gender, although attraction data were not collected in this study. Existing research indicates the merit of considering sexual minority populations as defined by both identity and behavior, as these dimensions of sexual orientation are not always overlapping and can reveal nuance in understanding the populations [1, 8]. For this study, we use the term SMW to include both women who self-identify as gay, lesbian or bisexual, and/or women who have sex with women and men (WSWM). We specifically state SM-FSW by identity or by behavior when differentiating between the groups.

Structural vulnerabilities are both direct and indirect drivers of HIV [9–13]. Housing insecurity has known associations with sexual and physical victimization, substance use, exacerbated mental and physical health conditions [14–16], HIV risk [17–19], and poor HIV outcomes [20, 21]. Violence against women, in all its iterations, has a substantial impact on poor health, physical and sexual injury, substance use, mental health, and the acquisition of sexually transmitted infections (STI), including HIV [22–27].

FSW confront a combination of structural vulnerabilities, which both limit their ability to reduce harm and hinder their access to health services, including HIV prevention, thereby increasing their HIV and STI risk [12]. Globally, FSW lifetime prevalence of experiencing violence in the workplace ranged from 45 to 75% [27]; street-based sex workers experience particularly high levels of violence, including physical, verbal, and sexual abuse [28–30].

Additionally, FSW face various HIV risk drivers. Street-based sex work and drug use are intricately linked in many settings in the USA, where women who use drugs often sell sex to support their drug habits and may engage in high-risk injection or sexual practices with partners [31]. The shared criminalization of both drug use and sex work compounds the increased HIV prevalence rates by preventing care-seeking and HIV risk reduction. Additional FSW vulnerabilities and HIV risk drivers are also driven by sex work criminalization and include endurance of egregious police behaviors; violence victimization and work environments; inability to access drug treatment; imprisonment, poverty, and housing instability; and stigma and discrimination [30, 32–36].

Compared to heterosexual women, SMW face exacerbated HIV risks. Rates of HIV are higher in SMW who have sex with high-risk men, use drugs, and/or are sex workers [3]. SMW are more likely to report higher rates of alcohol and other substance use and dependence [3, 37–39]. Bisexual women are at increased odds for sexual abuse, intimate partner violence (IPV), and physical violence compared to heterosexual women [4]. Further, young SMW experience higher rates of violence [40], are more likely to be engaged in survival sex work [2, 41], and have higher HIV risk-related sex work practices, such as inconsistent condom use and a greater number of clients, than their heterosexual counterparts [2].

Research is lacking regarding SM-FSW, a population that is doubly marginalized due to their sexual orientation and their profession. We examine the differences in structural vulnerabilities and HIV risk drivers between SMW, by identity and behavior, and their heterosexual counterparts among street-based FSW in Baltimore City, MD, USA.

## Methods

### Data source

Data analyzed in this paper were collected as part of a larger research study, the Sex workers And Police Promoting Health In Risky Environments (SAPPHIRE) study—a prospective cohort of women involved in street-based sex work recruited between April 2016 and August 2017 in Baltimore City, MD, USA. SAPPHIRE’s broad goal was to examine the role of the police in the HIV risk environment of street-based sex workers. A more detailed exploration of methods, including the study’s novel targeted sampling methods, are available elsewhere [42, 43]. Briefly, locations were selected through mapping of arrest data and information collected through Baltimore City Police Department ride-alongs. During 3–4-h recruitment periods, study staff approached single women who were visually soliciting clients. Eligibility criteria include the following: (1) age ≥ 15 years; (2) sold or traded oral, vaginal, or anal sex “for money or things like food, drugs, or favors”; (3) picked up clients on the street or at public places ≥ 3 times in the past 3 months; and (4) willing to undergo HIV and STI testing. The exclusion criterion was identifying as male or a man.

Participants completed a 50-min interviewer-administered computer-assisted personal interview survey. The survey included sections on demographics, housing, finances, sex work history, police encounters, incarceration, sexual and drug use behaviors, health service access, childhood abuse, and mental health. Participants were also tested for HIV and STI. Participants received a pre-paid $70 USD VISA gift card for completing the baseline survey. The study was approved by the Johns Hopkins Bloomberg School of Public Health Institutional Review Board.

### Study variables

Survey items in the SAPPHIRE study were theoretically informed by the risk environment framework and the theory of structuration as well as drawn from existing validated scales, our previous studies [5, 44], and the expertise of the SAPPHIRE Community Advisory Board, a group comprised of current and former sex workers.

#### Independent variables

SM status was defined separately by identity and behavior. Participants were asked if they identified as heterosexual/straight, gay/lesbian, or bisexual; this variable was dichotomized to heterosexual vs. non-heterosexual (gay/lesbian, or bisexual)^1^. Further, participants were asked about the gender of their sexual partners (vaginal or anal sex with a female client or regular partner in the past 3 months); this variable was dichotomized to heterosexual vs. non-heterosexual (WSWM).

#### Confounding variables

Age, race, and education were selected as control variables based on the literature and theoretical importance.

#### Potential dependent variables

Vulnerability variables included homelessness (past 3 months), arrest (lifetime), childhood abuse (sexual and physical), IPV (sexual and physical; lifetime), HIV, substance use (injection drug use [IDU], heroin, speedball, crack; past 3 months) and binge drinking (past 12 months)^2^, and sex work characteristic (minor at sex work entry, ever had a pimp/manager, years in sex work). Childhood (< 18 years old) abuse was defined as ever being pressured or forced into sexual intercourse or sexual touching (sexual), or being hit, punched, slapped, or otherwise physically hurt by someone causing marks or physical injury (physical). IPV items were derived from the Revised Conflict Tactics Scale [45] and were defined as either (1) being hit, punched, slapped, or otherwise physically hurt, or being threatened or hurt with a weapon (physical), or (2) being involuntarily pressured or forced into sexual intercourse (sexual).

### Sample

The analytic sample comprised 247 cisgender FSW. The sample excluded participants who identified as intersex (*n* = 1), did not have male or female clients in the past 3 months (*n* = 1), indicated being “confused” regarding sexual identity (*n* = 1), and had incomplete data on either the identity or behavior variables (*n* = 1).

### Statistical analyses

Bivariate differences between the independent variables (SM-FSW by identity and by behavior) and potential dependent variables (structural vulnerabilities and HIV risk drivers) were compared using Pearson’s chi-square tests for categorical variables and simple linear regression for the continuous variable (the age variable did not pass the Shapiro-Wilk normality test), with *p* < 0.100 indicating statistical significance.

Covariates significant *p* < 0.100 at the bivariate level were entered in the multivariable logistic regression models as dependent variables and SM status as independent variables. Multivariable models accounted for clustering within the zone of recruitment and controlled for age, race, and education. A total of 14 models are included. For example, homelessness was significant at the bivariate level for both classifications of SM status; for the multivariable model, two separate models were run for the same dependent variable: model 1 included control variables and SM status by behavior as independent variables and homelessness as dependent; model 2 included control variables and SM status by identity as independent variables and homelessness as dependent. This process was repeated for all covariates significant at the bivariate level. We report adjusted odds ratios (AOR) and 95% confidence intervals (CI). All analyses were conducted using Stata MP 15 software (StataCorp).

## Results

Roughly one quarter (25.5%) of participants reported SM status by identity and 8.5% by behavior (see Table 1). Of those who reported SM identity, 70% reported sex with men only and 30% reported sex with women and men in the past 3 months. Only 2 heterosexual-identified women reported sex with women.

**Table 1:** SM-FSW by identity and behavior (past 3 months)

		SMW by behavior		Total
		WSMO*	WSWM*	
SMW by identity	Heterosexual identified	182 (80.53%)	2 (9.52%)	**184 (74.49%)**
	Gay or bisexual identified	44 (19.47%)	19 (90.48%)	**63 (25.51%)**
	Total	**226 (100%)**	**21 (100%)**	**247 (100%)**

*WSMO-* women who report sex with men only, *WSWM-* women who report sex with women and men

*Past 3 months

The sample was 66% white, with a mean age of 35.7, and 53% reported having less than a 12th-grade education. Rates of current homelessness (62.8%), previous arrest (82.2%) and incarceration (69.8%), childhood violence (33.5% sexual, 42% physical), IPV (24.3% sexual, 47.5% physical), substance use (70.4% IDU, 24% binge drinking), depression (85.5%), and PTSD (60.8 %) were all high. HIV prevalence was 5.3%. Half of the sample (51.8%) had been engaging in sex work for more than 5 years (Table 2).

**Table 2:** SM-FSW characteristics by identity and behavior

	Total, 247 (100%)	Identity			Behavior		
		Heterosexual, 184 (%)	Gay/lesbian/bi, 63 (%)	*p* value	WSMO, 226 (%)	WSWM, 21 (%)	*p* value
Socio-demographics							
Age, mean (SD)	35.7 (9.0)	**36.3 (9.1)**	**33.8 (8.5)**	**0.06**	35.9 (8.9)	33.0 (10.5)	0.15
Race/ethnicity				0.16			**0.02**
White	163 (66.0)	126 (68.5)	37 (58.7)		154 (68.1)	9 (42.9)	
Black, Hispanic, others	84 (34.0)	58 (31.5)	26 (41.3)		72 (31.9)	12 (56.1)	
Relationship status				0.76			0.63
In a relationship or married	82 (33.3)	60 (32.8)	22 (34.9)		74 (32.9)	8 (38.1)	
Single	164 (66.7)	123 (67.2)	41 (65.1)		151 (67.1)	13 (61.9)	
Education- less than grade 12				0.18			
	131 (53.0)	93 (50.5)	38 (60.3)		**116 (51.3)**	**15 (71.4)**	**0.08**
Structural vulnerability							
Homeless*	155 (62.8)	**108 (58.7)**	**47 (74.6)**	**0.02**	**138 (61.1)**	**17 (81.0)**	**0.07**
Ever been arrested	203 (82.2)	149 (81.0)	54 (85.7)	0.40	186 (82.3)	17 (81.0)	0.88
Food insecure	134 (54.3)	99 (53.8)	35 (55.6)	0.81	121 (53.5)	13 (61.9)	0.46
Childhood sexual violence	80 (33.5)	56 (31.1)	24 (40.7)	0.18	71 (32.3)	9 (47.4)	0.18
Childhood physical violence	100 (42.0)	75 (41.7)	25 (43.1)	0.85	93 (42.1)	7 (41.2)	0.94
Lifetime physical IPV	115 (47.5)	**78 (43.3)**	**37 (59.7)**	**0.03**	105 (47.5)	10 (47.6)	0.99
Lifetime sexual IPV	60 (24.3)	41 (22.3)	19 (30.2)	0.21	54 (23.9)	6 (28.6)	0.63
HIV							
HIV-positive	13 (5.3)	11 (6.0)	2 (3.2)	0.39	13 (5.8)	0 (0.0)	0.26
Substance use							
Injection drug use*	174 (70.4)	**124 (67.4)**	**50 (79.4)**	**0.07**	159 (70.4)	15 (71.4)	0.92
Heroin injection*	160 (64.8)	116 (63.0)	44 (69.8)	0.33	147 (65.0)	13 (61.9)	0.77
Speedball injection*	56 (22.8)	**37 (20.2)**	**19 (30.2)**	**0.10**	51 (22.7)	5 (23.8)	1.00
Smoked crack*	206 (83.4)	153 (83.2)	53 (84.1)	0.86	189 (83.6)	17 (81.0)	0.75
Binge drinking**	84 (34.0)	**57 (31.0)**	**27 (42.9)**	**0.09**	**73 (32.3)**	**11 (52.4)**	**0.06**
Sex work-related characteristics							
Minor (< 18 years) at sex work entry	53 (21.5)	**30 (16.3)**	**23 (36.5)**	**< 0.001**	**41 (18.1)**	**12 (57.1)**	**< 0.001**
Ever had a pimp/manager	23 (9.3)	**13 (7.1)**	**10 (15.9)**	**0.04**	**18 (8.0)**	**5 (23.8)**	**0.02**
> 5 years in sex work	128 (51.8)	93 (50.5)	35 (55.6)	0.49	114 (50.4)	14 (66.7)	0.15

*Past 3 months

**Past 12 months

### SM-FSW by identity

Compared to heterosexual FSW, SM-FSW by identity were younger (33.8 vs. 36.3; *p* = 0.06) and reported elevated rates of homelessness (74.6% vs. 58.7%; *p* = 0.02), physical IPV (59.7% vs. 43.3%; *p* = 0.02), IDU (79.4 % vs. 67.4%; *p* = 0.07), and speedball injection (30.2% vs. 20.2%; *p* = 0.10). SM-FSW by identity were more likely to report being a minor at age of sex work initiation (36.5% vs. 16.3%; *p* < 0.001) and ever having a pimp/manager (15.9% vs. 7.1%; *p* = 0.04) (Table 2).

In adjusted models (Table 3), SM-FSW by identity were at greater odds than heterosexually identified FSW to have reported homelessness (aOR = 1.89, 95% CI 1.11–3.23, *p* = 0.02), physical IPV (aOR = 2.00, 95% CI 1.29–3.12, *p* = 0.002), injection drug use (aOR = 2.29, 95% CI 1.17–4.48, *p* = 0.02), binge drinking (aOR = 1.99, 95% CI 1.49–2.67, *p* < 0.001), and beginning sex work as a minor (aOR = 2.60, 95% CI 1.50–4.51, *p* = 0.001).

**Table 3:** Multivariable associations between SM-FSW status and structural vulnerabilities—GEE logistic regression with variance clustering for zone (adjusted odds ratios and 95% confidence intervals)

	aOR	95% CI	*p* value
Injection drug use*			
SMW by identity	**2.29**	**1.17–4.48**	**0.016**
SMW by behavior	1.49	0.41–5.42	0.544
Injection speedball*			
SMW by identity	1.72	0.79–3.72	0.238
SMW by behavior	1.08	0.33–3.54	0.327
Binge drinking**			
SMW by identity	**1.99**	**1.49–2.67**	**< 0.001**
SMW by behavior	**2.80**	**1.77–4.44**	**< 0.001**
Homeless*			
SMW by identity	**1.89**	**1.11–3.23**	**0.019**
SMW by behavior	**2.64**	**1.25–5.57**	**0.011**
Physical IPV***			
SMW by identity	**2.00**	**1.29–3.12**	**0.002**
SMW by behavior	1.03	0.41–2.61	0.95
Ever had a pimp			
SMW by identity	2.23	0.94–5.33	0.070
SMW by behavior	**3.36**	**1.94–5.74**	**< 0.001**
Minor at sex work entry (age < 18)			
SMW by identity	**2.60**	**1.50–4.51**	**0.001**
SMW by behavior	**4.82**	**2.68–8.68**	**< 0.001**

aOR adjusted for respondent age, race, and education

*Past 3 months

**Past 12 months

***Lifetime

### SM-FSW by behavior

Compared to heterosexual FSW, SM-FSW by behavior were less likely to be white (42.9% vs. 68.1%; *p* = 0.02) and more likely not to have completed high school (71.4% vs. 51.3; *p* = 0.08). SM-FSW by behavior reported elevated levels of homelessness (81% vs 61%; *p* = 0.071), binge drinking (52.4% vs. 32.3%; *p* = 0.06), being a minor at age of sex work initiation (57.1% vs. 18.1%; *p* < 0.001), and ever having a pimp/manager (23.8% vs. 8%; *p* = 0.02) (Table 2).

In adjusted models (Table 3), SM-FSW by behavior were at greater odds than women who have sex with men only (WSMO) to have reported homelessness (aOR 2.64, 95% CI 1.24–5.57, *p* = 0.01), binge drinking (aOR = 2.80, 95% CI 1.77–4.44, *p* < 0.001), ever having a pimp (aOR = 3.36, 95% CI 1.94–5.74, *p* < 0.001), and beginning sex work as a minor (aOR = 4.82, 95% CI 2.68–8.68, *p* < 0.001).

## Discussion

This study is one of the first to explore the association between SM status, by identity and by behavior, and structural vulnerabilities and HIV risk among street-based FSW. While FSW are characterized by significant structural vulnerabilities and HIV risk, we found these outcomes exacerbated among SM-FSW, by both identity and behavior, including substance use, homelessness, physical IPV, entering sex work as a minor, or ever having a pimp. Our results have important implications for improving HIV prevention and overall health promotion initiatives to better meet the needs of SM-FSW and to drive additional research endeavors.

The overall trends in our study tell a story of increased vulnerability among SM-FSW, with differences between the identity and behavior groups regarding IDU and physical IPV, both of which were significantly higher for SM-FSW by identity but not behavior. These differences support considering multiple dimensions of sexual orientation among the high-risk groups of women, similar to previous work [1, 46]. Findings also suggest that identity-related factors, such as stigma and discrimination, might explain some heightened risk. The minority stress theory [47, 48] posits that people with marginalized identities face exacerbated stressors from stigma and discrimination that accrue over time and lead to long-term health deficits. Coping with minority stress may occur through a range of maladaptive mechanisms, including IDU and increased violence exposure.

The results indicate an increased likelihood of two substance use behaviors, IDU and binge drinking, corroborating existing literature regarding substance use disparities among a general SMW population. The HIV risk implications of IDU are well-documented [49, 50]. The increased rates of IDU among SM-FSW by identity (79.4%) differed from a 2015 Baltimore study focusing on female IDUs, who also reported high rates of sex exchange (35%) [1]. SMW are more likely to report binge drinking compared to their heterosexual counterparts [51–53]. A relationship has been found between SM-related rejection and increased alcohol use [54]. Aside from the direct health consequences, binge drinking is associated with transactional sex engagement among a general population of women [55, 56] and unprotected sex, sexual violence, and STI rates among FSW [55, 56]. Minority stress may explain elevated substance use as a means of coping. HIV prevention programs targeting FSW must work in collaboration with harm reduction and substance use treatment programs, with a special attention to SMW. We are not aware of programs currently doing this work. Further, as sexual minority women have been largely considered “not at risk” of HIV, they have not benefited from mainstream HIV prevention programming, albeit few target FSW in the USA.

The exacerbated likeliness of homelessness among SM-FSW points to the depth of structural vulnerability in this population. Homelessness is associated with a range of deleterious health outcomes, including elevated rates of HIV [57]. SMW, regardless of sex work involvement, have increased rates of homelessness [58] due to family conflict, stigma, and sexual abuse [59, 60]. Homeless SM youth are more likely to engage in lifetime survival sex and sex with an HIV-positive partner [61]. Taken together, these data underscore the importance of focusing on SM status when designing HIV prevention and housing security interventions for FSW. These points are particularly relevant for working with younger populations.

SM individuals experience higher rates of violence victimization than heterosexuals across the life course [40, 62]. Elevated rates of IPV are associated with race, socio-economic status [63], and expectations of prejudice and discrimination [64, 65]. Internalized homophobia, resulting from minority stress, is linked to IPV perpetration and victimization [66]. We found that SM-FSW by identity have higher odds of physical IPV compared to their heterosexual counterparts, aligning with previous research among a general SMW population [67, 68], indicating unique vulnerability related to sexual minority status. Further, barriers to existing IPV services include a limited understanding of SM IPV, stigma, and systemic inequities [69]. SM-FSW face unique and exacerbated IPV vulnerabilities further intensified at the intersections of additional marginalized identities (i.e., race, class). Violence prevention programs should increase the utilization of a sexual orientation lens in their work, which could include gender and sexuality sensitivity training for staff, sexual minority-targeted outreach and programming, and policy-level work incorporating the needs of SM-FSW.

The increased odds of early sex work engagement and having a pimp among SM-FSW, found above, have not previously been explored. Research investigating health outcomes of domestic sex-trafficked victims^3^ regardless of sexual orientation indicates significantly worse health outcomes than that of non-trafficked women engaging in sex work, including physical health, suicidal ideation, substance use disorders, and healthcare deprivation [70, 71]. Sex work experiences are associated with drug and/or alcohol addiction, co-occurring illnesses, suicidal ideation, workplace violence, and condom non-use and failure [70, 71]. Having an abusive pimp is associated with co-occurring illnesses, poor overall physical and mental health, and suicidal ideation [70]. In sum, SM-FSW face increased risk vulnerability, particularly related to violence victimization throughout the life course, and may, again, be tied to minority stress and stigma related to their sexual minority status.

We found higher rates of SM status by both identity and behavior among our sample of FSW, compared to women in a generalized population study. A quarter (25.5%) of our sample identified as lesbian or bisexual, and 8.5% reported sex with women and men in the last 3 months. These data are much higher than women in a general population sample (1.5% lesbian/bisexual identified, 2% lifetime both-gender behavior) [37], indicating a relationship between SM status and sex work engagement, also found elsewhere [72]. LGBT young people frequently face family rejection, which impacts substance use, social support access, mental health, and homelessness [61, 73]. Coupled with the higher likelihood of sex work engagement risk behaviors among SM-FSW, we hypothesize that early life adversity related to SM status drives sex work engagement among SMW.

### Limitations

These results should be considered in light of several limitations. First, the construction of the SM variables was limited. The sexual behavior question was asked within a 3-month time period, so would not have captured women who have sex with other women, but who have not done so recently. Further, the category includes both regular partners and clients, which may mask differences in women who have sex with various genders in the working context vs. in their personal life. Data were not available regarding attraction, so this dimension of sexual orientation was not examined. Second, to address small cell sizes, we utilized a binary race variable, white and people of color (POC); therefore, we were unable to assess the differences among POC. More research is needed to better examine the differences across race, a known HIV vulnerability driver, and sexual orientation. Further, we were limited in looking at specific SM identities. Bisexual women are known to have a unique risk profile from their lesbian counterparts [74]; future research may identify further disparity risk profiles among SM-FSW subgroups. Last, this analysis was conducted only among the cisgender women in the SAPPHIRE study. The assumption of binary gender excludes gender non-binary individuals, who may have unique risk profiles.

## Conclusion

Our results indicate that SM-FSW experience exacerbated structural vulnerabilities and HIV risk drivers, as compared to their heterosexual FSW counterparts, a group that already experiences high rates of structural and HIV-related vulnerabilities. SM-FSW are a high-risk group within a high-risk group. This multiple marginalization is likely further exacerbated when considering young women. FSW-focused interventions must consider the unique needs of SMW and develop trauma-informed multi-level harm reduction programs, which can simultaneously address the complex constellation of structural and HIV risk faced by these women. This is particularly important for younger women, for whom opportunities to intervene early may protect against exacerbated vulnerability across the life course. People in all social service programming (i.e., substance use, housing support, violence prevention, sex worker advocacy, anti-trafficking, and sex work alternatives) need to understand SMW’s unique exacerbated risks. More research surrounding SM-FSW unique experiences is necessary to inform customized HIV prevention and overall health promotion interventions for this population.

## Data Availability

The datasets generated and/or analyzed during the current study are not publicly available in order to protect participant confidentiality but are available from the corresponding author on reasonable request.

## Abbreviations

AOR: Adjusted odds ratios
CI: Confidence intervals
FSW: Female sex workers
HIV: Human immunodeficiency virus
IDU: Injection drug use
IPV: Intimate partner violence
LGBT: Lesbian, gay, bisexual, transgender
POC: People of color
PTSD: Posttraumatic stress disorder
SAPPHIRE: Sex workers And Police Promoting Health In Risky Environments study
SM: Sexual minority
SMW: Sexual minority women
SM-FSW: Sexual minority female sex workers
STI: Sexually transmitted infections
WSWM: Women who have sex with women and men
WSWO: Women who have sex with women only

## Footnotes

1 Two additional response options, queer and same gender loving, received no responses.

2 Additional drugs were tested but were not significant at the bivariate level (results not shown).

3 Women who entered the sex trade due to coercion, fraud, etc. and/or before the age of 18.
